# BioModels: ten-year anniversary

**DOI:** 10.1093/nar/gku1181

**Published:** 2014-11-20

**Authors:** Vijayalakshmi Chelliah, Nick Juty, Ishan Ajmera, Raza Ali, Marine Dumousseau, Mihai Glont, Michael Hucka, Gaël Jalowicki, Sarah Keating, Vincent Knight-Schrijver, Audald Lloret-Villas, Kedar Nath Natarajan, Jean-Baptiste Pettit, Nicolas Rodriguez, Michael Schubert, Sarala M. Wimalaratne, Yangyang Zhao, Henning Hermjakob, Nicolas Le Novère, Camille Laibe

**Affiliations:** 1 European Molecular Biology Laboratory, European Bioinformatics Institute (EMBL-EBI), Wellcome Trust Genome Campus, Hinxton, Cambridge CB10 1SD, UK; 2 Computing and Mathematical Sciences, California Institute of Technology, Pasadena, CA 91125, USA; 3 Babraham Institute, Babraham Research Campus, Cambridge CB22 3AT, UK; 4 GlaxoSmithKline, Gunnels Wood Road, Stevenage, Hertfordshire SG1 2NY, UK

## Abstract

BioModels (http://www.ebi.ac.uk/biomodels/) is a repository of mathematical models of biological processes. A large set of models is curated to verify both correspondence to the biological process that the model seeks to represent, and reproducibility of the simulation results as described in the corresponding peer-reviewed publication. Many models submitted to the database are annotated, cross-referencing its components to external resources such as database records, and terms from controlled vocabularies and ontologies. BioModels comprises two main branches: one is composed of models derived from literature, while the second is generated through automated processes. BioModels currently hosts over 1200 models derived directly from the literature, as well as in excess of 140 000 models automatically generated from pathway resources. This represents an approximate 60-fold growth for literature-based model numbers alone, since BioModels’ first release a decade ago. This article describes updates to the resource over this period, which include changes to the user interface, the annotation profiles of models in the curation pipeline, major infrastructure changes, ability to perform online simulations and the availability of model content in Linked Data form. We also outline planned improvements to cope with a diverse array of new challenges.

## INTRODUCTION

BioModels is a portal to the modelling world which provides access to a wealth of mathematical representations of biological process, as well some of the tools with which they can be manipulated and simulated. Since the development of models has become an increasingly common and important tool in the analytic arsenal of both data and experimental scientists, it has become even more important to enable model sharing and reuse within and between different communities of users. The first step necessary to facilitate useful sharing and exchange of mathematical models was a standard vehicle through which they could be encoded. This was achieved with the advent of machine-readable, description languages such as Systems Biology Markup Language (SBML) (1) and CellML (2) to encode models. Simultaneously, there was a need to create repositories to store and distribute these models.

BioModels (3,4) serves a multitude of functions: models can be submitted to allow retrieval by other interested parties (sharing), can be downloaded for verbatim reuse (reference), or can be used as a scaffold to which refinements can be introduced (extension). Furthermore, the content of BioModels can also be regarded as providing reusable parts, from which components (submodels) can be extracted and aggregated to generate models of novel composition, usable for purposes beyond the intent of the original model itself.

**Figure 1.**
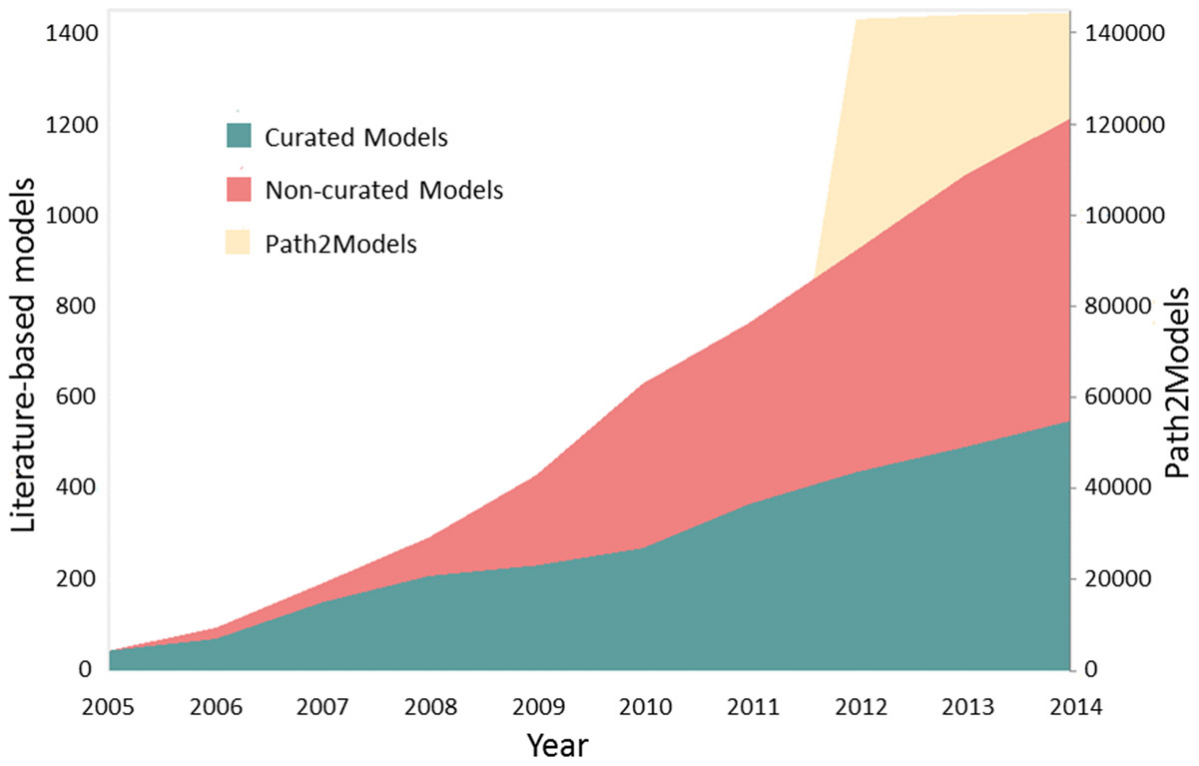
Growth of BioModels since its first release. The number of models submitted to the repository has increased significantly over the 10 years since BioModels’ launch in 2005. The number of literature-based models (green and pink areas) has grown from 20 (release 1, April 2005) to over 1200 (release 28, September 2014), an increase of more than 60-fold increase. A further 144 282 models (yellow area) are provided as part of the Path2Models branch (from release 22, May 2012). Note the different y-axis scales, where the left axis is used for literature-based models (green and pink areas) and the values for Path2Models (yellow area) use the right y-axis.

Over the 10-year period since the first release of BioModels, the modelling field has burgeoned as evidenced by the increased volume of model submissions to the repository. The original release of BioModels in 2005 contained around 20 models, while the latest release (release 28, September 2014) boasts well over 1200 literature-based models, and over 140 000 models generated through the automated processing of pathway resources (Figure 1). This 60-fold growth, in literature-based models alone, is but one of the challenges faced by BioModels. During the same period, models have become more complex (more components, more relationships or interactions between components), and are being generated from more disciplines, many of which have their own preferred formats. This article summarizes many of the changes to BioModels since its original release, many of which have been required to meet the ever-changing needs of the growing community of users.

## BIOMODELS CONTENT

BioModels content is divided into two major branches, which are handled quite differently. The first branch, available since the first release of BioModels, is concerned with literature-based models. The second branch was introduced much more recently, and is concerned with models that are generated by automated processing of pathway resources. To avoid confusion, these are considered separately in the subsequent sections.

### Literature-based models

BioModels accepts models encoded in SBML and CellML formats, but the internal, native, format of the resource is SBML. Upon submission, authors are provided a unique model identifier which can be referenced in submitted journal articles. The objective of BioModels is to provide public access to the model as soon as possible following publication of the corresponding article. Additionally, to facilitate the peer review process, advance access to submitted models can be provided to reviewers. A number of scientific journal publishers recommend model submission to BioModels as part of their author submission guidelines. These include journals from the EMBO press, Public Library of Science (PLoS), Royal Society of Chemistry (RSC), BioMed Central (BMC), ScienceDirect and FEBS Publishers.

Prior to being made publicly available, models submitted to the resource are subjected to annotation and curation processes. During the annotation phase, individual model components are cross-referenced to external database records and ontology terms to unambiguously identify them. For example, model components that are proteins may be cross-referenced to a protein database such as UniProt (5). These cross-references were historically made using a Uniform Resource Name (URN), which required the use of web services to retrieve further information on the cross-referenced entity. This system has been superseded by the use of resolvable Identifiers.org Uniform Resource Identifiers (URIs) (6), allowing users to directly view such annotations in a web browser. Individual models submitted to the resource are evaluated for compliance with the MIRIAM guidelines (7) to ensure not only that the model contains all information required to reproduce simulation results, but also to provide adequate provenance information.

The curation phase is focused on reproducibility of published results, using the information contained within the model. If this is demonstrable, a curation figure displaying representative simulation result(s) is attached to the model with comments from the curator on what protocol was used to regenerate the published result. If curators cannot reproduce the published results, the model submitters or authors are contacted for further information. Depending on the outcome of this processing, models are divided into one of two main categories: curated models which are fully MIRIAM compliant; and non-curated models which have not been curated.

### Path2Models: models generated by automated means

There exist a number of pathway data resources which provide a qualitative representation of key biochemical processes which take place within a cell. The Path2Models (8) effort was driven by the desire to systematically and automatically transform these representations into quantitative ones, where previous such efforts were largely *ad hoc* and manually intensive. It entailed the processing of many commonly used pathway resources such as the Kyoto Encyclopaedia of Genes and Genomics (KEGG) (9), BioCarta (http://www.biocarta.com/) and MetaCyc (10) to generate basic models, which could be supplemented with kinetic information, either fetched from resources such as SABIO-RK (11) or produced *ab initio* using heuristics from the pathway structure.

A clearly separated branch was created in BioModels to host the results of this effort. These models are significantly different to those already hosted in BioModels: they are not published in journals, are not peer-reviewed, are annotated by automated processes, and are not subjected to curation. The Path2Models branch was introduced in BioModels with release 22 (May 2012).

These models have been classified into three different types (based on the resource from which they are generated) and are made available to browse under the headings ‘metabolic’ (quantitative, kinetic metabolic pathways), ‘non-metabolic’ (qualitative, logical non-metabolic pathways) and ‘whole-genome metabolism’ (genome-scale metabolic network reconstructions). Alternatively, it is possible to identify relevant models through a ‘taxonomy’ interface, where models are displayed in an alphabetical listing, by species.

Since the initial release of this set of models, similar efforts (12) have been carried out, such as with the Nature Pathway Interaction Database (PID) (13), which are hosted within the Path2Models set. In total, this set describes biological processes for in excess of 2600 organisms, and provides models in SBML format, as well as SBGN-ML (14) format in some cases. Annotations for this branch, using resolvable URIs as with literature-based models, are generated by automatic processing of the information provided by the original resources. While every precaution has been taken to ensure that the annotations are appropriate, it should be borne in mind that they have not been validated by a curator.

## BIOMODELS FEATURES

**Figure 2.**
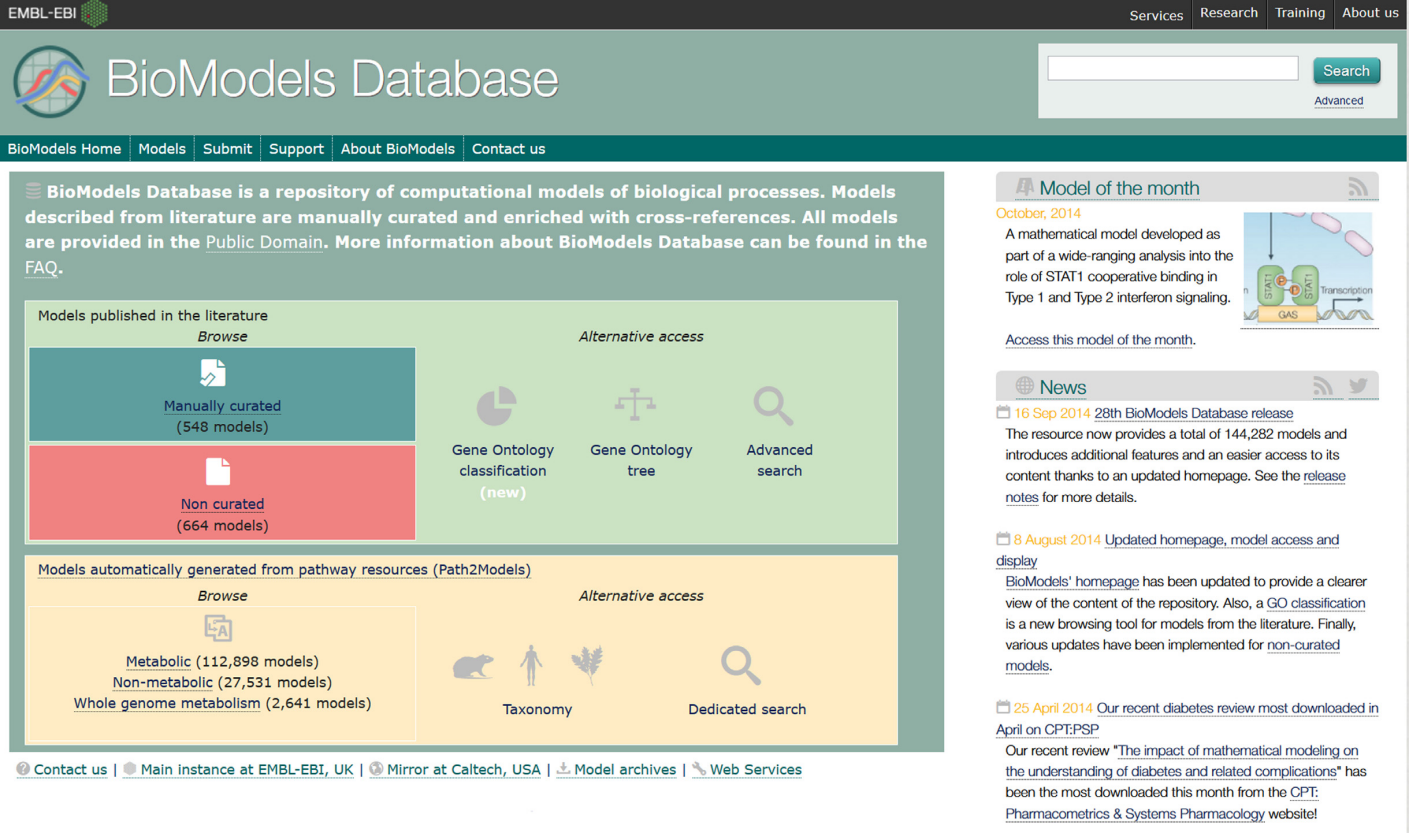
BioModels homepage. Due to the growing complexity and number of models, BioModels provides an improved interface to assist users in locating their target model(s). Models can be browsed through a simple listing, viewed through a GO categorization chart, or listed using an expandable GO tree view. Models generated from pathway resources (Path2Models) are listed by category, or alternatively may be viewed thought a taxonomic listing. Search mechanisms are available for both types of models.

Over the years, the web interface to BioModels has seen a number of rounds of improvement culminating in the current version (Figure 2). With the growing number of models and their components, it has become increasingly difficult for a user to efficiently retrieve their target models. This issue will be exacerbated by the growing number of models generated by automated processing of genomic information. BioModels now provides a number of ways to browse models, a much improved search interface, and also permits the programmatic search and download of models through Web Services (15).

### Retrieval of models

**Figure 3.**
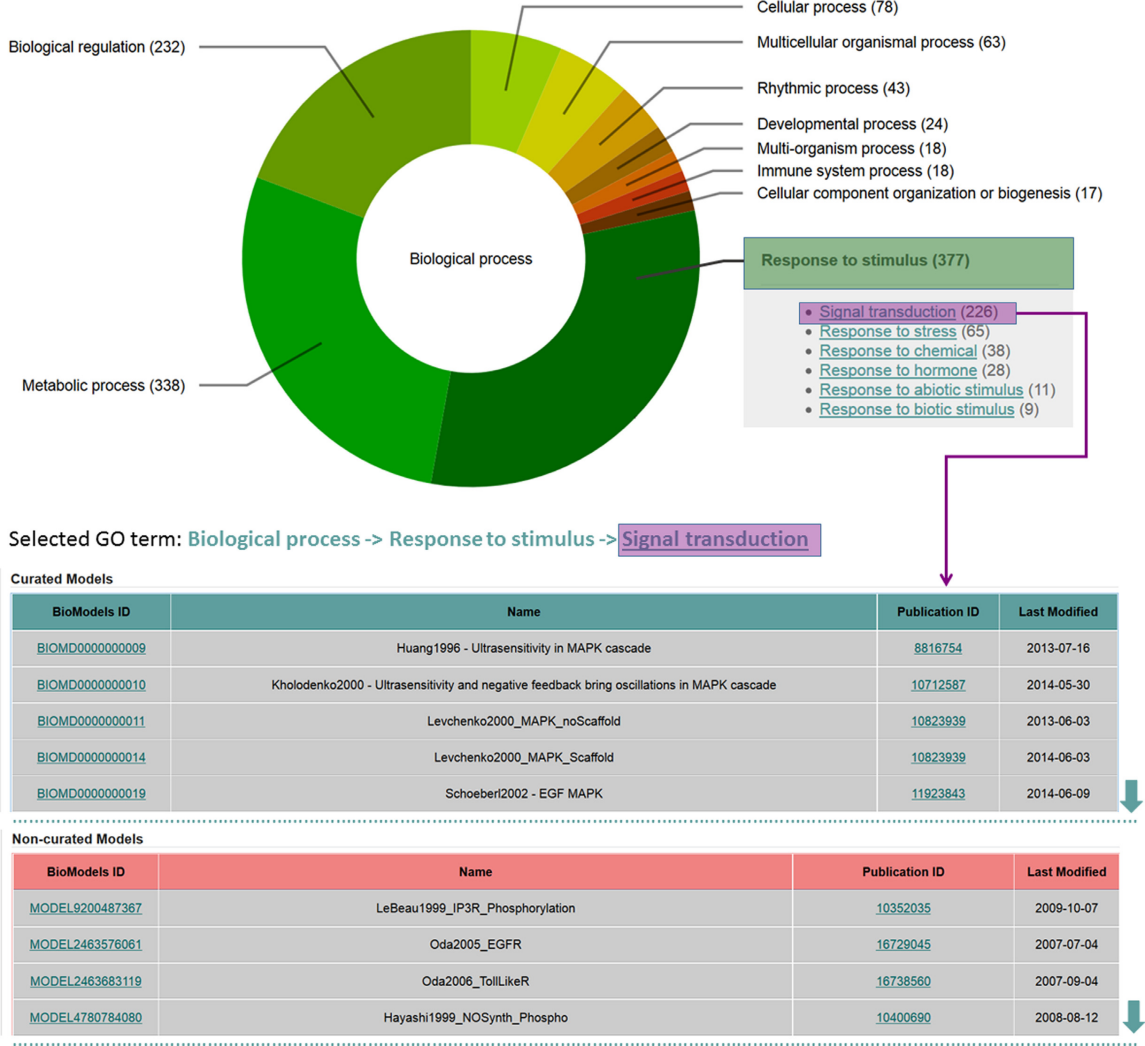
BioModels’ content and categorization. Literature-based models are divided into categories based on the GO term assigned to them. In total, there are four GO terms attached to each model, each successively more specific. Access to this categorization is provided through a dynamic chart, where the main GO branch ‘Biological process’ acts as the first level category. The second level categories are displayed as segments. Selection of one individual (here ‘Response to stimulus’) expands the display (bounded inset) to specify the precise number of models available in each corresponding third level category. Selection of second or third level categories provides a listing of all the corresponding models, distinguishing curated and non-curated models. Here, a part of the list of curated and non-curated models corresponding to the third level term ‘Signal transduction’ is shown.

Model level annotations provide information about the model as a whole, specifying the relevant biological process using Gene Ontology (GO) (16) terms, state the taxonomic range to which the model is applicable, and provides model lineage information, when available, to describe from which other model(s) or publication(s) the model was derived. With recent releases, model level annotation has been extended to include non-curated models, where originally only curated models were guaranteed to be annotated to at least this level, but also included annotations at the ‘physical entity’ and often at ‘math’ and ‘parameter’ levels. These annotations can be used to restrict queries through the advanced search feature. Furthermore, a generic categorization has been implemented through the clustering of individual GO terms, allowing aggregation of related models. This allows users to ‘drill down’ from a general category into more specific ones, whilst providing a full list of models in that category at each stage. This categorization allows visualization of models through a dynamic chart encompassing all models from the literature (Figure 3). An alternative way to browse curated models is provided through an interactive tree view of GO terms.

A simple search can be launched with a keyword from any page within BioModels, the results of which are presented as a list of models within which the keyword was found. The results page is divided, potentially, into three segments corresponding to models found in the curated category (literature-based branch) of models, the non-curated category (literature-based branch) and the Path2Models branch. The advanced search, applicable only to the literature-based models, makes use of model level annotations (including author and publication information), information stored in individual files (for instance the ‘notes’ elements in SBML files), and cross-reference information stored in the model. It also allows the selection of models which contain specific annotations to one or more specified resources. To improve the relevance of information returned to the user, the search results are subjected to post-processing. For instance, taxonomical searches are expanded to account for the relationships between taxons; a search for ‘*mammalia*’ will also retrieve models annotated with ‘*Homo sapiens*’ and ‘*Mus musculus*’, due to the taxonomic relationship with the original query term.

### Model display, download and simulation

Once the model of interest has been identified, detailed information about the model, its components and, if appropriate, the mathematics that describe its behaviour can be found through the web interface. This information is organized into various tabs. The ‘Model’ display page provides model level information, including annotations such as authors and submitters of the model, as well as GO terms that describe the biological process in which the model is significant. The ‘Overview’ tab provides a comprehensive list of model constituents, where each link acts as a shortcut to the more detailed descriptions in the subsequent tab (in parentheses). This lists all model entities (Physical entities), parameter information (Parameters), and mathematical relationships between entities (Maths). The ‘Curation’ tab provides information on the process required to reproduce the simulation generated.

Each model may be downloaded in a variety of SBML levels and versions. It is also possible to download models in alternative forms, including human readable reports in PDF (17) and tool specific formats such as XPP (18), Octave (MatLab m-file; http://www.gnu.org/software/octave/), SciLab (http://www.scilab.org/) and Virtual Cell (VCML) (19)or other standards, such as BioPAX (http://www.biopax.org/).

Over time, BioModels has collected together many individual converters under a generic framework called the Systems Biology Format Converter (SBFC). This framework (http://sourceforge.net/projects/sbfc/) is implemented in Java and is available as a standalone program. It is used by BioModels to interconvert SBML into a variety of formats.

There are a variety of facilities, made available through an ‘Actions’ button, that can be executed from the model display page. These include the ability to view automatically generated images of the model network components, in either SVG or PNG format. It is also possible to run simulations for curated models directly on BioModels’ infrastructure. This feature allows the user to select the model species and the duration of the simulation, and provides numerical and graphical results. For some models, simulation can also be executed through JWS Online (20).

Besides the ability to download models individually, a bulk download of the repository's content is also available, with archives regenerated weekly and with each BioModels release. These are provided through the EBI FTP server (http://ftp.ebi.ac.uk/pub/databases/biomodels/releases/).

### BioModels-linked dataset

Linked Data is becoming an increasingly popular method to describe, expose and integrate biological data and is reliant upon RDF (Resource Description Format). This entails providing information as triples (subject-predicate-object), as a way to describe the relationship between individual entities, using controlled vocabularies.

In order to provide access to BioModels’ content to the rapidly growing semantic web community, BioModels data has been provided as a linked dataset (21). This entailed the generation of an RDF representation of the models in the repository. So far, this includes all literature-based models and ‘whole-genome metabolism’ models from Path2Models, comprising around 175 million triples with over 34 million cross-references. The Linked Dataset is stored using OpenLink Virtuoso, and the RDF files themselves are regenerated with each new release of BioModels. Individual RDF models are provided as part of the downloadable archives.

This work is carried out as part of an institute wide pilot study (22), with the dataset exposed through the BioModels SPARQL endpoint (http://www.ebi.ac.uk/rdf/services/biomodels/sparql). SPARQL allows construction of federated queries across multiple resources and facilitates data integration.

### Model of the month

BioModels features a regular ‘Models of the Month’ (http://www.ebi.ac.uk/biomodels-main/modelmonth), drawn from a subset of hosted models (literature-based models). The feature serves to showcase selected models from the repository, and is presented as a short article. It includes introductory material for the subject area of the model, and discusses the results and significance of model simulations. These articles are a valuable asset for teaching, and promote the accessibility of modelling for novices to the field.

One recent effort by the BioModels team was the ‘targeted curation’ of models related to diabetes and its related clinical complications (23). It is envisaged that more such ‘targeted curation’ activities will take place in the future, looking into clinically significant areas.

## CONCLUSION

The modelling landscape has changed significantly since the software infrastructure underlying BioModels was originally developed in 2005, giving rise to many new challenges. These include increased model size and complexity, incorporation of high throughput efforts into modelling workflows, and the emergence of new formats (24,25). For BioModels to progress in tandem with the modelling landscape, it is necessary to upgrade its underlying software infrastructure and continue providing state of the art models.

To this end, BioModels is leading the development of a new generic and modular infrastructure, Jummp (JUst a Model Management Platform), to facilitate efficient collaborative model development and curation. This requires implementation of appropriate model management and versioning capabilities which are not currently available in BioModels. In addition, this will allow BioModels to extend its scope by providing support for new formats, such as the developing COMBINE Archive (http://co.mbine.org/documents/archive), which bundles together all documents necessary to share the description of a model, together with those required to facilitate its reuse (including the reproduction of simulation experiments). Jummp is an open source project, and is currently hosted on Bitbucket (https://bitbucket.org/jummp/jummp/).

Simultaneously, we seek to improve user accessibility of the resource (search and interface) and to pro-actively enhance and collate modelling data within high impact domains (*via* targeted curation efforts) (23).

BioModels serves as a valuable tool for the scientific community, providing access to a diverse array of biologically and biomedically relevant models. BioModels’ content is provided under the terms of the Creative Commons CC0, Public Domain Dedication, meaning that all models available may be freely downloaded, used, modified and redistributed, by any user.
